# Integration of Metabolomics and Proteomics in Exploring the Endothelial Dysfunction Mechanism Induced by Serum Exosomes From Diabetic Retinopathy and Diabetic Nephropathy Patients

**DOI:** 10.3389/fendo.2022.830466

**Published:** 2022-03-25

**Authors:** Jing Yang, Dongwei Liu, Zhangsuo Liu

**Affiliations:** ^1^ Department of Ophthalmology, The First Affiliated Hospital of Zhengzhou University, Zhengzhou, China; ^2^ Research Institute of Nephrology, Zhengzhou University, Zhengzhou, China; ^3^ Henan Province Research Center for Kidney Disease, Zhengzhou, China; ^4^ Key Laboratory of Precision Diagnosis and Treatment of Chronic Kidney Disease in Henan Province, Zhengzhou, China; ^5^ Department of Integrated Traditional and Western Nephrology, The First Affiliated Hospital of Zhengzhou University, Zhengzhou, China

**Keywords:** metabolomics, proteomics, exosomes, diabetic nephropathy, diabetic retinopathy, endothelial dysfunction

## Abstract

**Background:**

The prevalence of diabetic microvascular diseases has increased significantly worldwide, the most common of which are diabetic nephropathy (DN) and diabetic retinopathy (DR). Microvascular endothelial cells are thought to be major targets of hyperglycemic damage, while the underlying mechanism of diffuse endothelial dysfunction in multiple organs needs to be further investigated.

**Aim:**

The aim of this study is to explore the endothelial dysfunction mechanisms of serum exosomes (SExos) extracted from DR and DN (DRDN) patients.

**Methods:**

In this study, human glomerular endothelial cells (HGECs) were used as the cell model. Metabolomics ultraperformance liquid chromatography-tandem mass spectrometry (UPLC-MS/MS) and proteomics tandem mass tag (TMT)-based liquid chromatography-tandem mass spectrometry (LC-MS/MS) together with bioinformatics, the correlation analysis, and the joint pathway analysis were employed to discover the underlying mechanisms of endothelial dysfunction caused by patient’s SExos.

**Results:**

It can be assumed that serum exosomes extracted by DRDN patients might cause endothelial dysfunction mainly by upregulating alpha subunit of the coagulation factor fibrinogen (FIBA) and downregulating 1-methylhistidine (1-MH). Bioinformatics analysis pointed to an important role in reducing excess cysteine and methionine metabolism.

**Conclusion:**

FIBA overexpression and 1-MH loss may be linked to the pathogenicity of diabetic endothelial dysfunction in DR/DN, implying that a cohort study is needed to further investigate the role of FIBA and 1-MH in the development of DN and DR, as well as the related pathways between the two proteins.

## 1 Introduction

Diabetes has become much more common during the last few decades all throughout the world. Diabetes’ global occurrence has risen substantially from 108 million in 1980 to 422 million in 2014, according to epidemiological data, and diabetes is anticipated to become the seventh leading cause of death by 2030 (1–4). The major lethal causes for diabetic patients are diabetic macrovascular and microvascular complications that are derived from the diabetes-induced endothelial dysfunction. Because their failure to downregulate the glucose transport rate results in intracellular hyperglycemia when glucose levels are high, microvascular endothelial cells are regarded to be the major targets of hyperglycemic damage (4, 5). High blood sugar level is considered to induce the microvascular endothelial dysfunction in which diabetic nephropathy (DN) and diabetic retinopathy (DR) are the most common disorders. According to the epidemiological type-2 diabetes surveys in Chinese major cities, DN and DR account for 39.7% and 31.5% of diabetic microangiopathy, respectively (4).

It is recognized that retinopathy and nephropathy occur simultaneously in long-term diabetes. As an important structural and overlapping determinant of disease development, the relationship between kidney disease and retinal disease has been discussed, and the term “renal-retinal syndrome” has been recognized by the public (6, 7). The polyol pathway is involved in the development of the two disorders, as are increased advanced glycation end products (AGEs) synthesis, increased expression of the AGE receptor and its activating ligands, activation of protein kinase C isoforms, overactivity of the hexosamine pathway, and other pathways (8–10). Additionally, the systemic inflammation induced by the albuminuria-impaired kidney is considered to accelerate the clinical course of microvascular complications in both the eye and the kidney (11–17). In this sense, the underlying mechanisms of hyperglycemic injury for microvascular endothelial cells require further investigation.

In type 2 diabetes (T2D) patients with reduced glomerular endothelial fenestrae, albuminuria and a drop in glomerular filtration rate (GFR) are significantly connected (18–21). Multiple causes contribute to glomerular endothelial cell (GEC) dysfunction, including increased permeability of GECs, stimulation of endothelial apoptosis, glycocalyx breakdown, and poor cross communication between endothelial cells and other renal cells (e.g., podocytes) (22–24). GEC injury is responsible for the occurrence of microalbuminuria, which is the early event of DN (22). Microalbuminuria is also a sign of endothelial dysfunction in the kidneys and throughout the body (25, 26). The retina is harmed by chronic hyperglycemia, which compromises the blood–retina barrier, resulting in extracellular fluid accumulation in the macula, as well as thickening of the capillary basement membrane and increased deposition of extracellular matrix (ECM) components (27–29). By upregulating angiogenic factors [e.g., vascular endothelial growth factor (VEGF)] over time, chronic retinal microvasculature damage induces capillary non-perfusion and retinal ischemia (14, 30–34). DR increases the risk of life-threatening systemic vascular disorders regardless of vision loss (35, 36).

Endothelial dysfunction is linked to diabetic microvascular problems due to a deficiency in angiogenesis, increased endothelial permeability, increased leukocyte adhesion, and reduced nitric oxide action (11). In the process of diabetes, the diffused endothelial dysfunction appears in multiple organs, indicating the existence of potential factors that induce endothelial cell injuries in the circulation. Exosomes are nanoscale membranous vesicles (30–100 nm) and enriched in specific proteins, lipids, nucleic acids, and glycoconjugates. There are abundant exosomes in blood, and they are involved in various cellular activities. For instance, they are responsible for remodeling the ECM and then releasing contents into recipient cells for the purpose of transmitting signals and molecules to target cells and organs. This pathway of vesicle transportation plays crucial roles in various disease developments, such as diabetes mellitus (DM) and diabetic microvascular diseases (37). DM patients had significantly larger quantities of extracellular vesicles (EVs) in their circulation than euglycemic control participants, according to a previous study (38). In addition, serum exosomes (SExos) from diabetic db/db mice severely impair the aortic endothelial cell functions from non-diabetic db/m+ mice, indicating the changes in the composition of SExos (39). We employed quantitative proteomics and metabolomics to examine the differential proteins and metabolites produced by exosomes in human glomerular endothelial cells (HGECs) to better understand the molecular mechanism of exosomes in diabetic macrovascular endothelial dysfunction.

## 2 Materials and Methods

### 2.1 Cell Culture

ScienCell Research Laboratories (PS-4000, San Diego, CA) provided the HGECs, which were grown at 37°C and 5% CO_2_ in endothelial cell medium (ECM; Sciencell Research Laboratories), which contained 10% fetal bovine serum (FBS, Gibco), 100 U/ml penicillin, and 100 mg/ml streptomycin. Cell passages ranging from 2 to 5 were used in these studies.

### 2.2 Patients and Samples

From January to November 2020, the enrolled patients were admitted to Zhengzhou University’s First Affiliated Hospital. Serum samples for the exosome’s isolations were obtained from 20 healthy volunteers and 20 patients diagnosed with DR and DN. All of the samples were frozen at -80°C. The ethics committee of Zhengzhou University’s First Affiliated Hospital gave its approval to this study (2021-KY-0872-002). Prior to participation in the study, all patients signed a written informed permission form.

### 2.3 Exosome Experiments

#### 2.3.1 Serum Exosome Isolation and Identification

SExos were isolated as previously reported (40). In order to eradicate cells and cellular debris, the collected serum had been centrifuged for 15 min at 3,000g for 15 min. The exosome separation was done with ExoQuick, a fast-acting exosome precipitation solution (System Biosciences, CA, USA). The exosome suspension was dissolved in phosphate-buffered saline (PBS) and kept at -80°C for future use. The exosomes were identified using transmission electron microscopy (TEM; Tecnai G2 Spirit 120KV). The exosome particle size and concentration were assessed at VivaCell Biosciences using the nanoparticle tracking analysis (NTA) method and the related software ZetaView 8.04.02 with the ZetaView PMX 110 (Particle Metrix, Meerbusch, Germany). The isolated exosome samples were diluted in 1× PBS buffer to assess particle size and concentration (Biological Industries, Israel). At 11 different sites, the NTA measurement was obtained and analyzed. The ZetaView system was calibrated using polystyrene particles with a diameter of 110 nm. Temperatures were maintained between 24°C and 26°C.

#### 2.3.2 Exosome Labeling

To suspend the exosomes obtained from patients’ serum, a mixture of 100 L PBS and 1 ml PKH67 (Sigma, in Diluent C) was utilized. After 5 min at room temperature, exosome labeling was halted by adding 3 ml of 1% bovine serum albumin, and the colored exosomes were extracted using the ExoQuick exosome precipitation solution. Here, 10 g of exosomes were introduced to HGECs after suspension in basal media. The cells were cultured for 6, 12, and 24 h at 37°C before being washed and fixed at room temperature. The addition of 4′,6-diamidino-2-phenylindole (DAPI; Sigma) was used to stain the nuclei for 10 min. The stained cells were observed using an Olympus confocal microscope (Japan).

### 2.4 Western Blot

Before being lysed in radioimmunoprecipitation assay buffer (RIPA) buffer, the cells were collected and rinsed in cold PBS (Solarbio, Beijing, China). At 4°C, the whole-cell lysates were centrifuged for 15 min at 12,000 rpm. Protein concentrations were determined using the bicinchoninic acid method. Sodium dodecyl sulfate–polyacrylamide gel electrophoresis (SDS-PAGE) was used to separate the proteins, which were then transferred to a nitrocellulose membrane. In the PBS containing 5% evaporated milk and 1% Tween-20, the membranes were blocked for 1 h before being treated overnight at 4°C with primary antibodies dissolved in PBS containing 1% Tween-20. The primary antibodies were anti-CD9 (Abcam, Hong Kong, China; # ab92726), anti-CD63 (Abcam, ab216130), anti-TSG101 (Sino Biological, Beijing, China; #102286-T38), anti-CD31 (Zen-Bioscience, Chengdu, China; #383815), anti-von Willebrand factor (vWF), (Abcam, #ab154193), anti-intercellular adhesion molecule 1 (ICAM-1) (Abcam, #ab171123), anti-vascular cell adhesion protein 1 (VCAM-1) (Abcam, #ab134047), and anti-Glyceraldehyde-3-Phosphate Dehydrogenase (GAPDH) (Goodhere, Hangzhou, China; #AB-P-R 001). The blots were then treated with the appropriate secondary antibodies on the second day: horse radish peroxidase (HRP)-labeled goat anti-rabbit immunoglobulin G (IgG) (Dingguo Changsheng, Beijing, China; #IH-0011) and HRP-labeled goat anti-mouse IgG (Dingguo Changsheng, Beijing, China; #IH-0011). The Odyssey two-color infrared laser imaging system (LICOR) or HRP-based chemiluminescence analysis was used to detect the signals. Image J/Fiji and GraphPad Prism 9.0 were used to analyze the grayscale of the Western blot (WB) bands (GraphPad Software, Inc., La Jolla, CA, USA).

### 2.5 Immunofluorescence

The immunofluorescence (IF) assays were conducted as described before (41). The HGEC lines were seeded on collagen-coated glass and cultured in ECM medium overnight at 37°C in a humidified 5% CO_2_ atmosphere. The cells were rinsed twice with PBS before being fixed with 4% formaldehyde and permeabilized with 0.2% Triton X-100. The cells were treated with fluorescein Alexa-Fluor 488-conjugated secondary antibodies for one night at 4°C after being blocked with 1% BSA for 30 min and photographed using a fluorescence microscope (Leica, Wetzlar, Germany). Tissues were treated overnight at 4°C with primary antibodies, then with secondary antibodies Goat Anti-Rabbit IgG H&L fluorescein isothiocyanate (FITC) (Abcam, #ab7086) pre-absorbed, Goat anti-Mouse IgG (H+L) cross-adsorbed, Alexa Fluor 555, and finally imaged using a confocal microscope (Olympus, Japan) and scanned using a slide scanner Pann (3DHISTECH, Hungary).

### 2.6 Proteomics Study

#### 2.6.1 Protein Extraction

Enzymatic hydrolysis was performed for 300 mg of each sample. After diluting dithiothreitol (DTT) to 100 mM, the sample was heated for 5 min and then cooled to room temperature. After that, the sample was given 200 ml of ultrasound-assisted (UA) buffer (8 M urea, 150 mM Tris-HCl, pH 8.0). The mixture was evenly mixed before being transferred to a 10-kD ultrafiltration centrifuge tube and centrifuged for 15 min at 12,000g. After adding 15 ml of UA buffer to the sample, it was centrifuged for 15 min and the filtrate was discarded. The sample was then incubated at room temperature for 30 min in the dark before being centrifuged at 12,000g for 10 min after being added 100 ml of 50 mM iodoacetamide (IAA) (in UA) and shaken at 600 rpm for 1 min. Samples were centrifuged twice at 12,000g for 10 min each time after being washed twice with 100 ml UA buffer. The material was then rinsed twice with NH_4_HCO_3_ buffer (100 ml) and centrifuged for 10 min at 14,000g. The sample was then treated with trypsin (20 mg trypsin in 40 ml NH_4_HCO_3_ buffer), agitated at 600 rpm for 1 min, and incubated at 37°C for 16–18 h. The material was centrifuged at 12,000g for 10 min after the collection tube was changed. After adding the trifluoroacetic acid (TFA) solution to the filtrate to a final concentration of 0.1%, the sample was desalinated with a C18 cartridge. The samples were quantified using OD280.

#### 2.6.2 Tandem Mass Tag (TMT)-Based Liquid Chromatography-Tandem Mass Spectrometry (LC-MS/MS)

Metware Biotechnology Co., Ltd., completed the TMT-based LC-MS/MS identification and data normalization analysis (Wuhan, China). The protein samples were diluted 6-fold and underwent enzyming and desalting. The peptide samples were diluted to 1 g/l on-board buffer in a 5-L container, and the scanning mode was set to 120 min. The peptides were scanned in the sample with a mass-to-charge ratio of 350–1,500. After preparing mobile phase A solution [98% water, 2% acetonitrile (ACN), 0.1% formic acid (FA)], B solution (98% ACN, 2% water, 0.1% FA), pre-column (300 m0.5mm, 3 m), analytical column (3 m, 75 m150 mm; Welch Materials, Inc), and spray voltage, peptides separated by liquid phase were ionized by the nanoESI source and transferred into the tandem mass spectrometer Q-Exactive (Thermo Fisher Scientific, San Jose, CA, USA). The main parameters are set as follows: 320°C of the ion transfer tube temperature, 350–1,500 m/z of scanning range, 60,000 of primary resolution, 3e6 of C-Trap, 80 ms of inject time (IT), 15,000 of secondary resolution, 1e5 of C-Trap, 100 ms of IT, 28 of CE, 1.0e4 of threshold intensity, and dynamic exclusion for 30 s. The raw data for mass detection (.raw) were generated, and the mass spectrometer data were retrieved and analyzed using MaxQuant 1.6.15.0.

#### 2.6.3 Protein Identification and Quantitative Analysis

The data from the mass spectrometer were collected and analyzed using MaxQuant 1.6.15.0. The following were the search parameters: TMT-10plex (Peptide Labeled) quantification type; Enzyme: Trypsin/P; Max. missed cleavages: 2; carbamidomethyl (C) is a fixed alteration. Protein quantification includes the following modifications: oxidation (M), acetyl (Protein N-term); First search MS/MS tolerance: 20 ppm; Main search MS/MS tolerance: 5 ppm; Include contaminants: TRUE; Decoy mode: revert; Peptides used for protein quantification: Razor; Protein FDR: 0.01; PSM FDR: 0.01; Include contaminants: TRUE; The sequencing was done using the UniProt Taxonomy Database (**Supplementary Material S1**). The mass spectrometry proteomics data have been deposited with the dataset number PXD030660 to the ProteomeXchange Consortium (http://proteomecentral.proteomexchange.org) *via* the iProX partner repository (42).

#### 2.6.4 Bioinformatics Methods

To learn more about the causes of SExo-induced endothelial dysfunction in DR+DN patients, researchers used the UniProt-GOA Database (http://www.ebi.ac.uk/GOA/) for Gene Ontology (GO) functional annotation and Kyoto Encyclopedia of Genes and Genomes (KEGG) pathway enrichment analysis. FDR 0.01 was chosen as the cutoff point. The UniProt Gene Ontology Annotation (GOA) database was used to enhance the GO annotation proteome, which was supplemented by InterProScan soft. Subcellular localization was predicted using Wolf PSORT software. The KEGG database was used to annotate protein pathways. The pheatmap function in the R package was used to build the clustering heat map. The bar graph and circos graph were created using the ggplot function from the R package -ggplot2.

#### 2.6.5 Protein Interaction Network Analysis

To use the IntAct (http://www.ebi.ac.uk/intact/main.xhtml) or STRING (https://string-db.org/) databases, first, get the gene symbol from the target protein’s sequence database. STRING is used to create protein–protein interaction (PPI) networks (a search tool for finding interacting genes). A confidence score of 0:4 and a maximum number of interactors of 0 are the cutoff conditions for this study. Then, using the Cytoscape software 3.2.1 (http://www.cytoscape.org/) platform, the interaction network of differentially expressed proteins was screened using cytoHubba based on high connectedness (43).

#### 2.6.6 Parallel Reaction Monitoring

The results of the TMT-based LC-MS/MS analysis were backed up by parallel reaction monitoring (PRM) analysis of chosen proteins. The distinctive peptides of the selected proteins were identified using TMT-based LC-MS/MS data, and only a few peptide sequences were chosen for PRM analysis. Trypsin was used to digest, reduce, alkylate, and absorb the selected proteins (100 mg). Through a C18 trap column (0.10 20 mm; 3 mm) and then a C18 column (0.15 120 mm; 1.9 mm), the collected peptide mixtures were injected into the mass spectrometer. A tabletop Orbitrap mass spectrometer (Q Exactive; Thermo Scientific) with a quadrupole mass filter was used to make the MS computation. Proteome Discoverer 1.4 was used to collect and evaluate raw data (Thermo Fisher Scientific). The false discovery rate (FDR) for proteins and peptides had been set to 0.01. Skyline 2.6 software was used for quantification and proteomic analysis. For the PRM analysis, three biological replicates were performed in each group.

### 2.7 Metabolomics Study

#### 2.7.1 Ultraperformance Liquid Chromatography-Tandem Mass Spectrometry (UPLC-MS/MS) Analysis

A Ultra High Performance Liquid Chromatography (UHPLC) system was used to perform the chromatographic separation (Waters Corp., Milford, MA, USA). ACQUITYTM UPLC BEH C18 (2.1 mm 100 mm, 1.7 m) was used to analyze all of the analytes. The column was held at a constant temperature of 35°C. The mobile phase was made up of solvent A (water and 0.1% formic acid) and solvent B (water and 0.1% formic acid) (acetonitrile). The injection volume and flow rate were set at 0.4 ml/min and 2 L/min, respectively. A Waters SynaptTM QTOF/MS spectrometer (Waters Corp., Milford, MA, USA) was coupled to a UHPLC system through an ESI source in both positive and negative ionization modes.

#### 2.7.2 Data Processing

Using the self-built target standard metware database, the qualitative analysis was performed based on the retention period of the detected chemical, the information of the precursor ion pairs, and secondary spectrum data (MWDB, containing business trade secrets). The metabolites were quantified using triple quadrupole mass spectrometry’s multiple reaction monitoring (MRM) mode analysis.

The metabolite content data were standardized using the unit variance scaling (UV) method. The R program (https://www.r-project.org/) was used to compare diverse samples and perform hierarchical cluster analysis (HCA) on the accumulation manner of metabolites. Principal component analysis (PCA; R function prcomp, parameters center = TRUE, scale = TRUE), orthogonal partial least squares discriminant analysis (OPLS-DA), and partial least squares discriminant analysis (PLS-DA) were used to analyze the data. The S-plot was made to see which marker components differed significantly between the groups. By scanning Internet databases such as KEGG, Human Metabolome Database (HMDB), and Metabolite Set Enrichment Analysis (MSEA), possible endogenous metabolites were discovered using accurate molecular masses, MS/MS fragments, and retention behavior. The R package MetaboAnalystR was used to run OPLS-DA (parameters, opls_log, “MeanCenter,” “S10T0,” ratio = FALSE, ratioNum = 20). Heat map was drawn by R package Heatmap [parameters na_col = ‘grey,’ rect_gp = gpar(col = cell.border.color, lwd = 1)]. R function cor was used to analyze Pearson’s correlation coefficient [parameter method = pearson; correlation graph was used to R package corrplot, parameters add = TRUE, type = ‘lower,’ method = ‘number,’ number.cex = mycex, number.digits = 2, diag = F, tl.pos = ‘n’].

### 2.8 Proteomics and Metabolomics Integration Analysis

The data were transformed to normalize the distributions. At least 20% of metabolites that were not presented in the samples were filtered out. Differential abundances of proteins among CON-EXO vs. DRDN-EXO individuals were calculated using the linear modeling R-package limma. Multiple testing corrections were applied using Benjamin and Hochberg FDR (P < 0.05 for significance). Volcano plots were plotted by R-package ggplot2. Pathway analysis was performed using the limma ROAST method. The analysis of the integrated proteomics and metabolite interaction network included the proteins related to 1-methylhistidine (1-MH) and excluded the isolated nodes.

### 2.9 Statistical Analysis

SPSS24.0 statistical software was used for the data analysis. The independent T test was used to compare the two groups, whereas the multivariate variance was used to compare the numerous groups. Statistical significance was defined as a P-value <0.05.

## 3 Results

### 3.1 Circulating Exosomes Could Influence Renal and Retinal Endothelial Cells and Cause Endothelial Dysfunction

**Figure 1:**
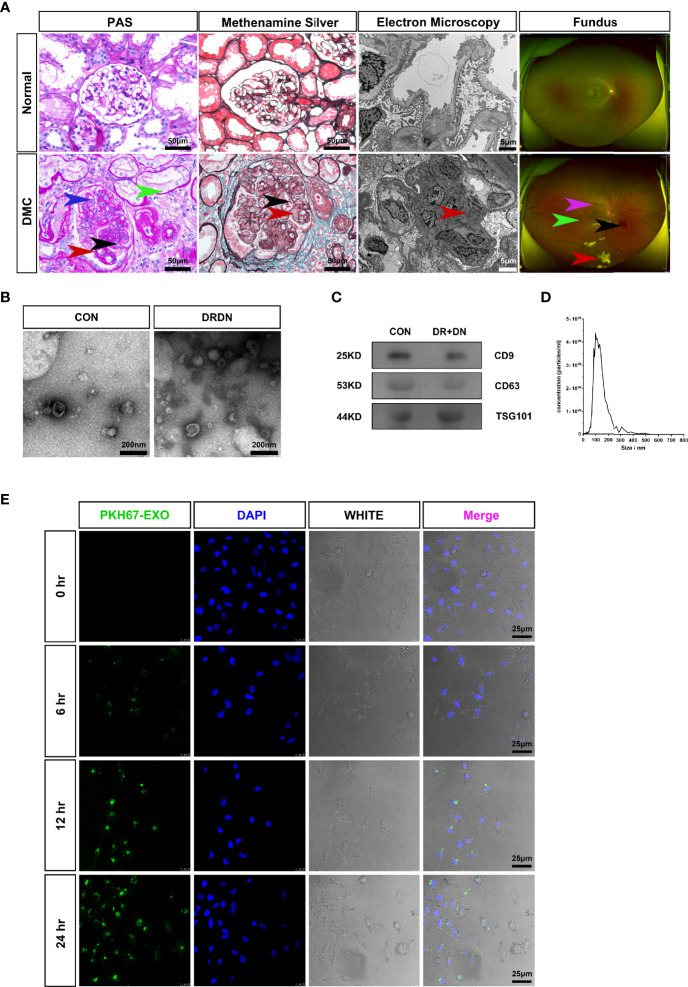
Serum exosomes from diabetic retinopathy and nephropathy patients could induce endothelial dysfunction. **(A)** The typical pathological kidney images and fundus images obtained from healthy people and a diabetic microvascular disease patient. **PAS**: The DMC biopsy sample showed proliferation and swelling of the endothelial cell (blue arrow), reduplication (double contour appearance) of the glomerular basement membrane (red arrow), mesangial expansion (black arrow), arteriolar hyalinosis (green arrow). Scale bar: 50 μm. **Methenamine silver:** Renal biopsy samples from the DMC patient show the swelling of the endothelial cells (red arrow) and reduplication (double contour appearance) of the glomerular basement membrane (black arrow). Scale bar: 50 μm. **Electron microscopy:** The slice from the DMC patient showed mesangial expansion (red arrow). Scale bar: 5 μm. **Fundus:** The fundus from DMC showed microhemangioma (green arrow), retinal exudates (red arrow), intraretinal hemorrhage (black arrow), and intraretinal microvascular abnormalities (IRMAs; violet arrow). **(B)** Identification of serum exosomes from CON patients and DRDN patients by transmission electron microscopy (TEM). Scale bar: 200 nm. **(C)** Western blotting was used to look for the exosomal markers CD9, CD63, and TSG101 in exosome samples. **(D)** Analysis of the size distribution of exosomes from patients using the NanoSight technology; the average size of serum exosomes was 107.5 ± 55.2 nm. **(E)** Exosome tracing experiment captured by confocal microscope. Blue for DNA dyed by DAPI and green for exosomes derived from DR+DN patients dyed by PKH67. HGECs were subjected to 6, 12, and 24 h of incubation with exosomes. Scale bar: 25 μm. PAS, Periodic acid–Schiff stain; DMC, diabetic microvascular complications; CON, healthy controls; DRDN, patients diagnosed with both diabetic retinopathy and diabetic nephropathy.

All of the patients in this study were separated into two groups: CON (healthy persons) and DR+DN (diagnosed as both DR and DN). **Figure 1A** shows the renal biopsy pathological and fundus images obtained from one DR+DN patient and one healthy participant, indicating that endothelial microvascular leakage of retina and kidney could occur simultaneously. In retina, the diabetic endothelial dysfunction led to several disorders such as the capillary wall dilatation (microaneurysms), the leakage (edema and hard exudates), and the rupture (hemorrhages). The dilation of the retinal capillary walls was an early physiological sign of microvascular malfunction. Diabetic endothelium damage manifested itself in the glomerulus as endothelial cell enlargement, glomerular basement membrane reduplication, mesangial expansion, and arteriolar hyalinosis. SExos from DR+DN patients were separated from the serum by ultracentrifugation and visualized by TEM after negative staining. Most of the SExos were less than 100 nm. A characteristic cup-shaped morphology was identified using TEM (**Figure 1B**). In the exosome fraction, Western blot analysis revealed strong CD9, CD63, and TSG101 (exosome markers) signals (**Figure 1C**). The Delta Nano C particle analyzer was used to characterize the characteristics of SExos in real time (ZetaView, Particle Metrix). The size of SExos was 121.8 ± 50.1 nm (mean ± SD) (**Figure 1D**). To investigate the communications between endothelial cells and SExos, we cocultured HGECs and PKH67-labeled SExos and photographed them using a confocal microscope at different time points (**Figure 1E**). The internalization of fluorescent exosomes was observed inside the endothelial cells, and it was found that exosomes had entered endothelial cells at 6 h, and the number of exosomes entering endothelial cells gradually increased over time.

**Figure 2:**
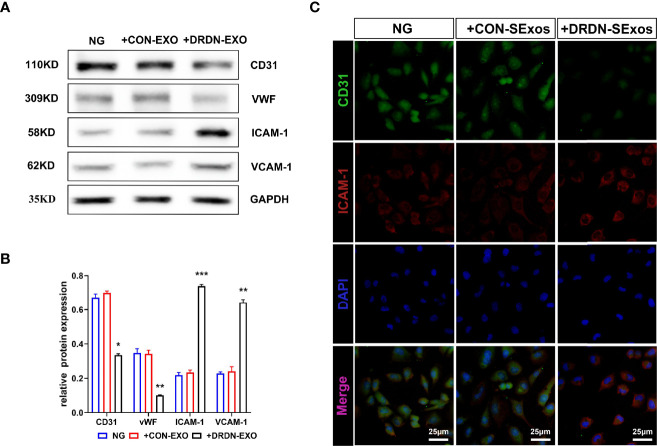
Serum exosomes from diabetic retinopathy and nephropathy patients could induce endothelial dysfunction. **(A)** Western blot analysis of CD31, vWF, ICAM-1, and VCAM-1 in HGECs treated with serum exosomes from healthy people and DR+DN patients. **(B)** Grayscale analysis of Western blot in HGECs treated with serum exosomes from healthy people and DR+DN patients, N = 3, *P < 0.05, **P < 0.01, ***P < 0.001. **(C)** The cellular immunofluorescence of HGECs stained with CD31 (green fluorescence) and ICAM-1 (red fluorescence). Scale bar: 50 μm.

In order to verify whether the serum exosomes extracted from DR+DN patients could induce injury effect on endothelial cells, we cultured HGECs with exosomes from DR+DN patients and healthy participants, respectively, and extracted cellular protein and mRNA 48 h later. Endothelial dysfunction indicators such as ICAM-1 and VCAM-1, as well as CD31 and vWF, were increased in HGECs treated with DR+DN SExos, whereas CD31 and vWF were dramatically downregulated (**Figures 2A, B** **)**. Cellular immunofluorescence confirmed that HGECs were injured when cocultured with the DR+DN-SExos (**Figure 2C**).

### 3.2 Analysis of Differentially Expressed Proteins by iTRAQ-Based Quantitative Proteomics

**Figure 3:**
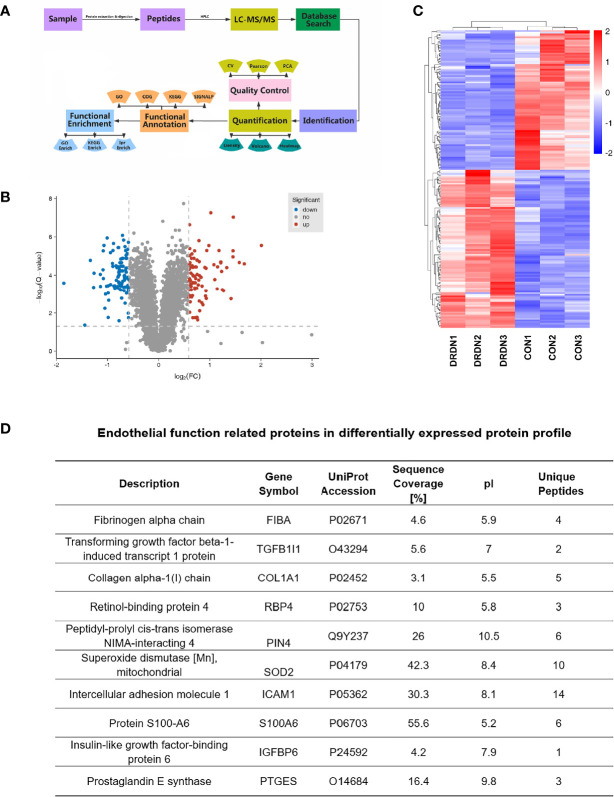
Global proteomics profiles of HGECs incubated with CON- and DRDN-SExo. **(A)** Technical route flowchart of metabolic profiles. **(B)** Volcano plot showing the relative content of differently between groups and the statistical differences. To create a volcano map, use log 2 (FC) as the abscissa and negative logarithm -log10 (adj. P-value) as the ordinate. There were 12 upregulated metabolites (red dots) and 8 downregulated metabolites (blue dots) (green dots). **(C)** Hierarchical clustering heat map of significantly differentially expressed proteins in all comparison groups. **(D)** Endothelial function-related proteins in differentially expressed protein profile.

To investigate the molecular mechanism that DR+DN-SExos induce endothelial cell dysfunction, HGECs were treated with SExos from healthy participants (CON group) and DR+DN patients (DRDN groups). The differentially expressed proteins between the CON groups and the DRDN groups were identified using iTRAQ-based proteomics (**Figure 3A**). Qualitative control analysis on the test samples was carried out (**Supplementary Figure S2**).

The FDR strategy’s BH (Benjamini and Hochberg) approach was utilized to further adjust the P-value of the multiple tests and obtain the adj. P-value. Finally, the differences of protein were screened out based on the differences between the multiple and the adj. P-value. Criteria for significant differences in expression are shown as follows: when the comparison group’s adj. P-value ≤0.05 and the fold change ≥1.5 (upregulation of expression) or fold change ≤0.67 (downregulation of expression), the expression is considered as a significant variety. **Figure 3B** depicted the volcano plots of proteins retrieved from healthy volunteers and DR+DN patients in HGECs treated with SExos. Results showed that after the treatment with 50 mM SExos from DR+DN patients for 48 h, a total of 185 differentially expressed proteins were identified in HGECs with 87 proteins upregulated (red dots) and 98 proteins downregulated (blue dots). The significant differences of expressed proteins between the groups were observed in the hierarchical clustering heat map as shown in **Figure 3C**. **Figure 3D** showed the proteins associated with endothelial dysfunction of the profile. Among the differentially expressed proteins, fibrinogen alpha chain (FGA) was the highest elevated (fold change: 4.024). Transforming growth factors beta 1 (TGFβ1) and Collagen Type I Alpha 1 Chain (COL1A1) have been linked to the diabetic endothelial-to-mesenchymal transition and have been shown to worsen diabetic glomerulosclerosis (44, 45). Circulating ICAM-1 levels are positively connected with albuminuria in individuals with type 1 diabetes (T1D) and T2D (46, 47). ICAM-1 is an endothelial damage protein marker (46). S100A6 is an important component of the PPP5C-FKBP51 axis, which protects the endothelium barrier from calcium entry-induced disruption (48).

### 3.3 Function Analysis of the Differentially Expressed Proteins in Human Glomerular Endothelial Cells Affected by Serum Exosomes Extracted From DR+DN Patients

**Figure 4:**
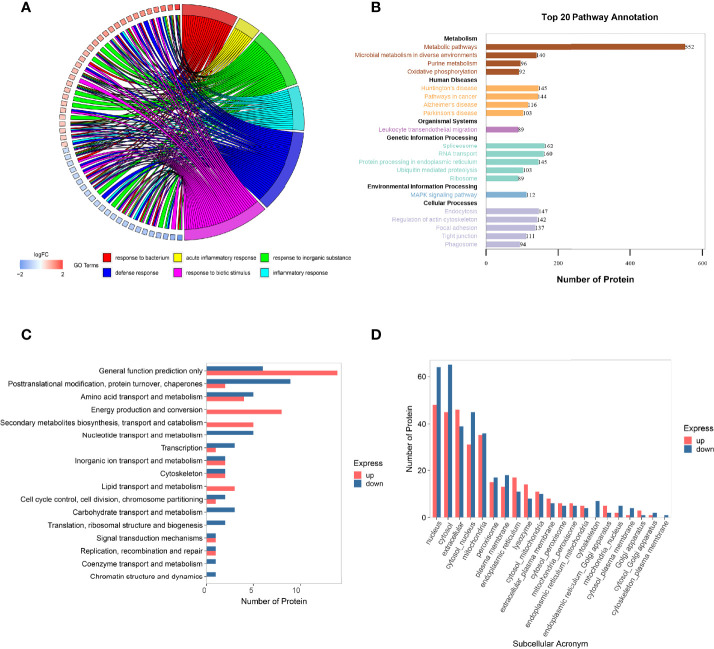
Function analysis of the differentially expressed proteins in HGECs affected by DR+DN patients’ SExos. **(A)** Circus plots of GO Classification Annotation of differential protein. **(B)** Pathway annotated result statistics chart. A histogram of the top 20 pathway names sorted by the number of annotated proteins. The abscissa represents the number of proteins, and the ordinate represents the annotated KEGG entry. **(C)** Differential protein COG classification annotation bar graph. Take differential protein as the analysis object, perform COG functional classification analysis, and compare the functional classification of upregulation and downregulation. **(D)** Statistics of subcellular localization results of differential proteins. Sub-cells are shown by the abscissa, while the number of proteins is represented by the ordinate.

The GO is an international classification system for gene functions that provides a systematic up-to-date standard vocabulary (controlled vocabulary) to thoroughly characterize the properties of genes and gene products in organisms. The GO annotation results of the differentially expressed proteins were shown in **Figure 4A** and **Supplementary Material S3**, revealing that the majority of these proteins are involved in bacterial response, defense response, acute inflammatory response, biotic stimulus, inorganic substance response, and inflammatory response.

The metabolic pathway, the focal adhesion pathway, the arginine and proline metabolism pathway, the phagosome pathway, the tight junction pathway, and the ECM–receptor interaction pathway were among the key pathways identified by the KEGG pathway annotation analysis (**Figure 4B** and **Supplementary Material S4**). The results of GO and KEGG analysis showed that the endothelial dysfunction mechanism of the DR+DN patients’ SExos was mainly affected by the metabolism processes and the multicellular communication processes and might have some correlations with the cell junction and inflammation processes.

The Cluster of Orthologous Groups of Proteins (COG) is a database that categorizes proteins into orthologous groups. The proteins constituting each type of COG are assumed to be derived from an ancestor protein and therefore are either orthologs or paralogs. The COG database was compared to the discovered proteins, and probable functions were anticipated using a functional categorization statistical analysis. **Figure 4C** and **Supplementary Material S5** showed the comparison of these statistical results with the COG database. The abscissa represents the number of proteins, and the ordinate represents the COG entry annotated. The upregulated proteins had a higher concentration of COG annotations in the categories of energy production and conversion, secondary metabolite biosynthesis transport and catabolism, amino acid transport and metabolism, and lipid transport and metabolism. The COG annotations for downregulated proteins were in the following order: posttranslational modification, protein turnover, chaperones, amino acid transport and metabolism, nucleotide transport and metabolism, and carbohydrate transport and metabolism. Other proteins were solely implicated in predicting general function and other metabolism processes. These findings suggest that SExos’ endothelial cell-damaging effects are linked to its ability to influence protein interactions and enzymatic processes.

It is only when proteins are transported to the correct position that the subcellular structures can perform different biological functions of organisms and participate in various cell activities. Therefore, it is very important to understand the protein subcellular location information for the organism activities. The subcellular location prediction was done using the Wolf PSORT website, and the statistical map of the prediction findings for differential subcellular positioning is displayed in **Figure 4D** and **Supplementary Material S6** (the abscissa represents the subcellular, and the ordinate represents the number of proteins). From the results, it can be seen that the subcellular localization is mainly concentrated in the area of the nucleus, cytoplasm, ECM, coexistence of nucleus and cytoplasm, and mitochondria. The functional analysis of proteomics profiling all pointed to the metabolic process.

### 3.4 Protein Interaction Network Analysis and Parallel Reaction Monitoring Validation of the Differentially Expressed Proteins in Human Glomerular Endothelial Cells Affected by Patients’ Serum Exosomes

**Figure 5:**
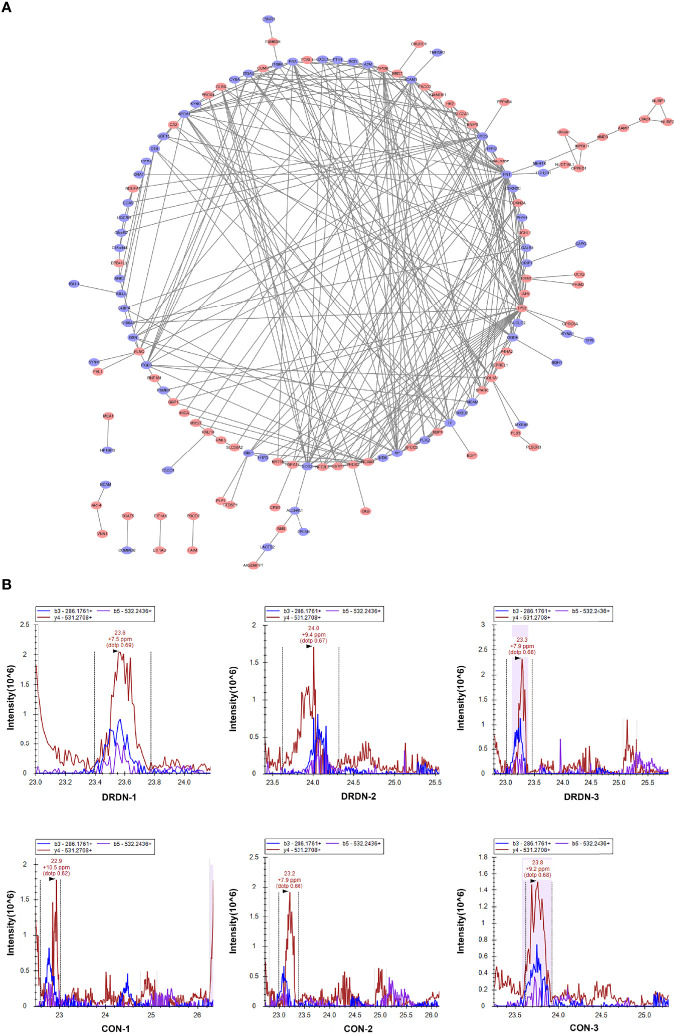
PPI analysis and PRM validation of significantly differentially expressed proteins in HGECs incubated with CON- and DRDN-SExo. **(A)** PPI analysis of differentially expressed proteins to describe putative links between the adhesion-related proteins tested. **(B)** PRM validation of FIBA protein in CON and DRDN groups.

To identify putative interactions between differentially expressed proteins, we created a PPI network using the STRING database version 9.0 (**Figure 5A**). Our analysis revealed that a majority of the screened proteins interact with one another directly or indirectly. We discovered 96 proteins linked to PPI networks in this PPI network. Fibronectin 1 (*FN1*) was the most important hub among these proteins, connecting with 19 others.

The most complicated networks contained *FN1*, COL1A1, and superoxide dismutase 2 (SOD2). As a result, these proteins should be given specific attention in follow-up investigations on DN dysfunction. SOD2 has been linked to diabetic microvascular problems such as DR and retinopathy. Among its related pathways are apoptosis and survival_anti-apoptotic action of nuclear ESR1 and ESR2 and oxidative stress. GO annotations related to this gene include identical protein binding and oxygen binding. Fibronectin, a protein involved in cell adhesion and migration, including embryogenesis, wound healing, and blood coagulation, is encoded by the *FN1* gene. *FN1* is linked to glomerulopathy with fibronectin deposits. The pathways of integrin cell surface contacts and VEGF signalling are linked. COL1A1 gives instructions for creating a part of type I collagen, the most abundant type of collagen in the human body. The VEGF signaling pathway is also linked to COL1A1.

FIBA, ICAM-1, and Peptidylprolyl Cis/Trans Isomerase, NIMA-Interacting 4 (PIN4), endothelial function proteins, were chosen and confirmed using PRM analysis to further investigate the target proteins. We explored that the levels of FIBA and ICAM-1 in SExos-induced HGECs were increased 1.37-fold and 1.21-fold, respectively, and the level of PIN4 was deceased 0.78-fold (**Table 1**). The validation results were compatible with the protein analysis data, even though the fold change values differed. **Figure 5B** showed the fragment ion peak distribution of FIBA peptides in all samples. Protein levels of FIBA were significantly upregulated in DR+DN-SExos-incubated HGECs.

**Table 1:** Confirmation of potential differentially expressed proteins related to endothelial function using PRM analysis.

Peptide	Protein	Gene Name	Fold Change (TMT)	Fold Change (PRM)
ALTDMPQMR	sp|P02671|FIBA_HUMAN	FGA	4.024	1.37
CQVEGGAPR	sp|P05362|ICAM1_HUMAN	ICAM-1	1.684	1.21
QGGDLGWMTR	sp|Q9Y237|PIN4_HUMAN	PIN4	0.666	0.78

TMT, Tandem mass tag-based liquid chromatography-tandem mass spectrometry; PRM, Parallel Reaction Monitoring; FGA,Fibrinogen alpha chain; ICAM-1, Intercellular Adhesion Molecule 1; PIN4, Peptidylprolyl Cis/Trans Isomerase, NIMA-Interacting 4.

### 3.3 Global Metabolic Changes in Human Glomerular Endothelial Cells Incubated With CON/DRDN-SExos

**Figure 6:**
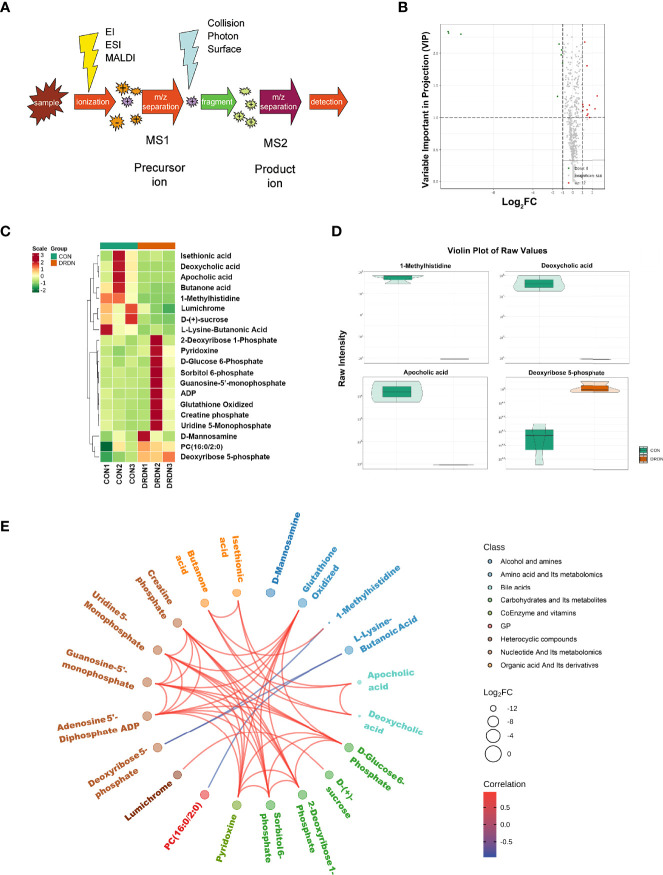
Metabolic profiles of HGECs incubated with CON- and DRDN-SExo. **(A)** Schematic diagram of the mass spectrometry multi-reaction monitoring mode. **(B)** The overall cluster diagram of the sample. The horizontal is the sample information, and the vertical is the metabolite information. Scale represents the obtained value after the standardization of the relative content of metabolites (the redder the color, the higher the content), and Group represents the grouping. **(C)** Volcano map of differential metabolite. A metabolite is represented by each point on the volcano map, with the green point representing the differential metabolite that is downregulated, the red point representing the differential metabolite that is upregulated, and the gray point representing the metabolite that is detected but not significantly different. The ordinate shows the VIP value, while the abscissa represents the logarithmic value of the relative content difference of the metabolites in the two groups. **(D)** Differential metabolite diagram in the form of a violin. The abscissa represents the sample, while the ordinate represents the differential metabolite’s relative content (original peak area). The interquartile range is shown by the middle box, the 95% confidence interval is represented by the thin black line that extends from it, the median is represented by the black horizontal line in the center, and the data distribution density is represented by the outside shape. **(E)** Differential metabolites are depicted as a chord diagram. The log2 FC value of the different metabolites is the outermost layer in the figure; the different colors represent the different classifications of the corresponding metabolites; the line represents the size of the Pearson correlation coefficient between the corresponding different metabolites, the positive correlation is represented by the pink line, and the negative correlation is demonstrated by the blue line; the line represents the size of the Pearson correlation coefficient between the corresponding different metabolites, the positive correlation is represented by the pink line, and the blue line represents negative correlation. By default, plot the differential metabolites with |r| > 0.8 and P < 0.05.

To further explore the metabolites and metabolic processes that DR+DN patients’ SExos induce endothelial dysfunction, the UPLC-MS/MS detection platform, combined with the univariate and multivariate statistical analysis methods, was used to study the metabolome differences between groups. Metabolite quantification was performed by using the MRM mode analysis of triple quadrupole mass spectrometry (**Figure 6A**). **Supplementary Figure S7** showed the overlay of the total ion current (TIC) graph using mass spectrometry detection for the quality control sample. The TIC curves for metabolite detection include a lot of overlap, indicating that signal stability is better when the mass spectrometer detects the same sample multiple times.

Based on the OPLS-DA results, the resulting variable importance projection (VIP) of the OPLS-DA model can be used to filter different metabolites between groups. Simultaneously, the univariate analysis P-value and fold change were employed to select other differential metabolites. When a metabolite met the conditions of fold change ≥2/≤0.5 and VIP ≥1, it was selected as a differential metabolite. The clustering heat map for different metabolites were shown in **Figure 6B**, with the ordinate representing the main differentially selected metabolites. The volcano plot shown in **Figure 6C** presented the relative contents of different metabolites and the statistical differences between the groups. There were 12 upregulated metabolites (red dots) and 8 downregulated metabolites (green dots). The details of differential metabolites between CON and DRDN groups had been demonstrated in **Supplementary Material S8**.

In order to show the data distribution density of different metabolites between groups, a violin chart showing the 4 differential metabolites which were the most significantly expressed was drawn with the ordinate representing the relative content of the different metabolites (**Figure 6D**). It was found that 1-MH, deoxycholic acid, apocholic acid, and deoxyribose 5-phosphate were the most changed metabolites after endothelial cells were treated with DR+DN patients’ SExos. The Pearson correlation analysis method (**Figure 6E**) was performed, and results showed that 1-MH, deoxycholic acid, and apocholic acid presented the positive correlations with butanone acid, while 1-MH presented the negative correlation with deoxyribose 5-phosphate.

### 3.4 Function Analysis of Differential Metabolites in Human Glomerular Endothelial Cells Treated With CON/DR+DN-SExos

**Figure 7:**
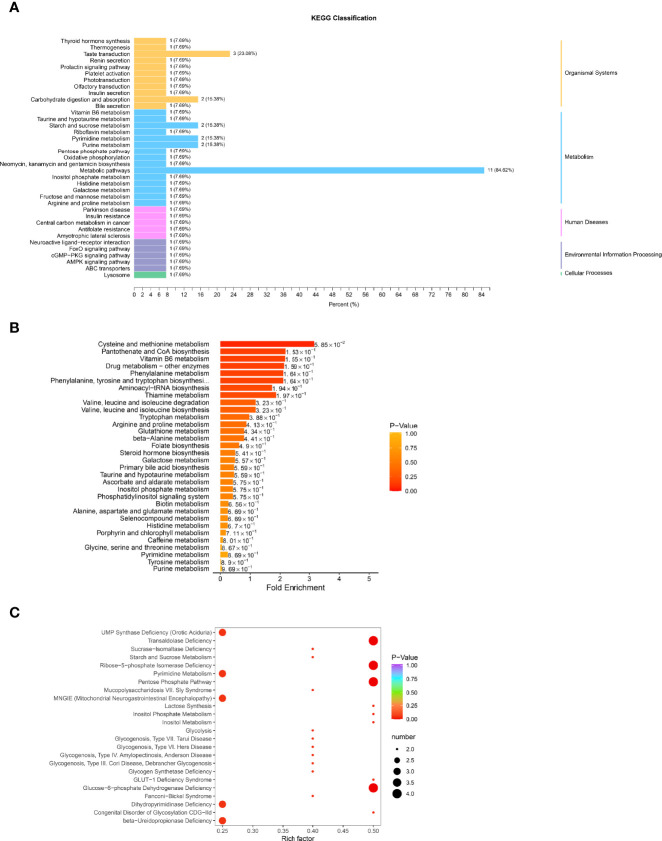
Function analysis of differential metabolites in HGECs treated with CON/DR+DN-SExos. **(A)** KEGG classification of various metabolites. The ordinate is the KEGG metabolic pathway, while the abscissa is the number of differential metabolites ascribed to the route and its fraction of the overall number of annotated metabolites **(B)** MSEA enrichment analysis chart. The ordinate indicates the name of the metabolic set, which corresponds to the P-value of the labeled metabolic set; the abscissa indicates the fold enrichment; the color indicates the P-value. **(C)** HMDB enrichment map for different metabolites. The abscissa is the rich factor associated with each path, whereas the ordinate is the path’s noun. The number of different metabolites enriched is represented by the size of the dot, and the color of the point denotes the P-value.

The possible metabolic processes for different metabolites were investigated by the KEGG pathway classification analysis. **Figure 7A** and **Supplementary Material S9** show the KEGG classification of the different metabolites, with the ordinate representing the KEGG metabolic pathway and the abscissa representing the number of different metabolites annotated to the pathway and their proportion to the total number of annotated metabolites. The metabolic pathways, which include starch and sucrose metabolism, pyrimidine metabolism, and purine metabolism, had the highest value of 85.62%, as expected. It is to be noted that the taste transduction is also one of the most important pathways. Because traditional enrichment analysis focuses on metabolites that are considerably upregulated or downregulated, it is easy to overlook metabolites that are not significantly differently expressed but have major biological implications. The MSEA was carried out using the MetaboAnalyst database (http://www.metaboanalyst.ca/). **Figure 7B** and **Supplementary Material S10** show the findings of the analysis. Cysteine and methionine metabolism, pantothenate and CoA biosynthesis, vitamin B6 metabolism, drug metabolism, and phenylalanine metabolism were the most important metabolic sets. The metabolic pathway enrichment was then performed simultaneously using the HMDB (https://hmdb.ca/) (**Figure 7C**, **Supplementary Material S11**). Transaldolase deficit, ribose-5-phosphate isomerase deficiency, pentose phosphate route, and glucose-6-phosphate dehydrogenase shortage were the most important metabolic pathways.

### 3.5 Integration Analysis Between Metabolomics and Proteomics

**Figure 8:**
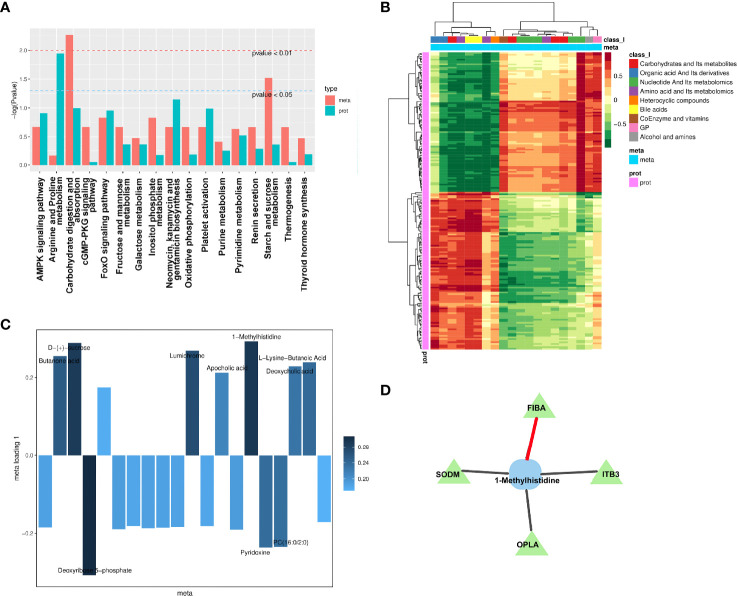
Integration analysis between metabolomics and proteomics. **(A)** Analysis of KEGG enrichment histogram of P-values. The metabolic route is indicated by the abscissa, the enriched P-value of differential metabolites is represented by red on the ordinate, and the enriched P-value of differential proteins is represented by green on the ordinate (P-value). **(B)** Correlation coefficient clustering heat map. Each row represents a protein, and each column represents a metabolite in the diagram. A positive correlation between genes and metabolites is represented by red, whereas a negative correlation between proteins and metabolites is represented by green. **(C)** Loading diagram of metabolites. The distance from each point in the figure to the origin or the height of the bar graph represents the correlation between matter and proteomics. **(D)** Diagram of a correlation network. Proteins are represented by green triangles, while metabolites are represented by blue squares. Positive correlation is shown by the black line, while negative correlation is represented by the red line.

According to the enrichment analysis results of differential metabolites and differential proteins, the histogram was drawn to show the degree of enrichment of pathways with differential metabolites and differential proteins at the same time (**Figure 8A**). The most noticeable pathway was the carbohydrate digestion and absorption, which was significant in the pathways with the coexistence of both differential proteins and differential metabolites. Moreover, the most significant pathway of differential protein in joint analysis was the arginine and proline metabolism that were reported to have specific associations with diabetic complications (49).

To investigate the link between metabolites and proteins, the Pearson correlation matrix was used (**Figure 8B**). Differential proteins expressed by endothelial cells treated by patient’s SExos showed a strong correlation with L-lysine-butanoic acid, 1-MH, deoxycholic acid, PC (16:0/2:0), apocholic acid, deoxyribose 5-phosphate, lumichrome, D-(+)-sucrose, and butanone acid.

Then, an O2PLS model was established based on all differential proteins and differential metabolites. The load diagram was used to preliminarily select the metabolites with high correlation and weight and screen out the metabolites that have the greatest impact on the differentially expressed proteins (**Figure 8C**). Results showed that 1-MH, D-(+)-sucrose, and deoxyribose 5-phosphate presented the greatest impacts. Then, the differential proteins and differential metabolites with correlations greater than 0.8 in all analysis pathways were selected for the network diagram (**Figure 8D**, **Supplementary Material S12**). It could be seen that 1-MH and FIBA had a positive correlation, while 1-MH had a negative correlation with superoxide dismutase [Mn] (SODM), integrin beta-3 (ITB3), and 5-Oxoprolinase (OPLA). Here, 1-MH was the most significant metabolite of endothelial cells processed by DR+DN patients’ SExos according to the metabolic analysis and was also the differential metabolite that was closely related to the differential proteins from the analysis above.

### 3.6 Overexpression of Fibrinogen Alpha Chain Exacerbated Endothelial Cell Injury in Human Glomerular Endothelial Cells

**Figure 9:**
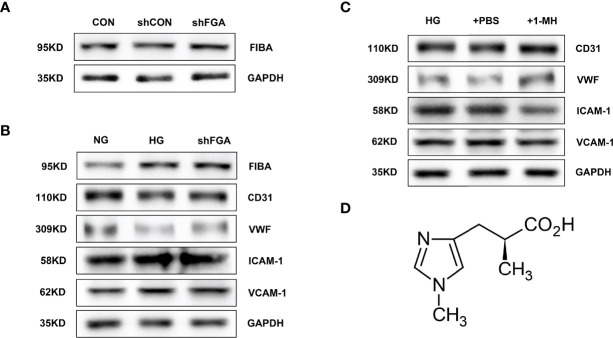
Overexpression of FGA exacerbated endothelial cell injury in HGECs; 1-MH rescued HGEC from injury induced by high glucose. **(A)** FIBA levels were considerably elevated in HGECs transplanted with FGA overexpressed shRNA compared to untransfected HGECs (CON group) and vector control cells (shCON group) (shFGA group). **(B)** The protein expressions of ICAM-1 and VCAM-1 were upregulated in shFGA and HG groups, while CD31 and vWF were significantly downregulated. **(C)** Compared with HG and +PBS groups, the protein expressions of ICAM-1 and VCAM-1 were significantly downregulated in HGECs with the increase of 1-MH content, while CD31 and vWF were upregulated. **(D)** The chemical structural formula of 1-MH.

Using adenovirus-mediated expression of FGA shRNA, we were able to successfully overexpress FGA (shFGA group). FIBA levels were considerably overexpressed in HGECs compared to untransfected HGECs (CON group) and vector control cells (shCON group) (**Figure 9A**). In order to verify whether overexpression of FGA could injure endothelial cells, we cultured HGECs with shFGA and high-glucose culture medium (HG group), respectively, and extracted cellular protein 48 h later. The protein expressions of endothelial dysfunction markers including ICAM-1 and VCAM-1 were upregulated in shFGA and HG groups, while CD31 and vWF were significantly downregulated (**Figure 9B**).

### 3.9 1-Methylhistidine Rescued Human Glomerular Endothelial Cells From Injury Induced by High Glucose

To further explore the effect of 1-MH on endothelial function, we added 1-methyl-L-histidine solution to HG-incubated HGECs (+1-MH group). The control group was HGECs incubated with HG culture medium added with the same volume of PBS (+PBS group). Compared with HG and +PBS groups, the protein expressions of ICAM-1 and VCAM-1 were significantly downregulated while CD31 and vWF were upregulated (**Figure 9C**). The increase of 1-MH could weaken the impairment effect of high glucose on HGECs. The chemical structural formula of 1-MH is shown in **Figure 9D**.

## 4 Discussion

DN and DR are the most common diabetic microvascular complications (2, 4, 5). However, the pathogenesis is still unclear. To date, there is rarely a proteomic profile or metabolome analysis involving diabetic SExos-induced endothelial dysfunction. We used TMT-based LC-MS/MS technology for the first time to characterize the whole proteome of HGECs incubated with SExos from DR+DN patients and UPLC-MS/MS analysis to investigate the differential metabolites. In this work, TMT technology was used to quantitatively analyze HGECs treated with SExos from healthy people or DR+DN patients, and a targeted metabolomic approach was applied to find 87 upregulated proteins and 98 downregulated proteins in HGECs treated with SExos from DR+DN patients. FGA was the most upregulated protein among the differentially expressed proteins. FGA encodes the alpha subunit of the coagulation factor fibrinogen (FIBA), which is a component of blood clot (50, 51). An important paralog of FGA is *FN1*, which is also the protein that interacts directly with most other proteins in PPI analysis. The researchers next used a broad-target metabolome study to detect 12 upregulated and 8 downregulated metabolites, with 1-MH showing the most significant changes in HGECs treated with SExos. A joint analysis of differential proteins and differential metabolites was conducted to further determine the role of target metabolites in DRDN and explore the link between differential proteins and differential metabolites. The results showed that 1-MH and FIBA may be correlated in the pathology of DR and DN. Overexpression of FGA worsened endothelial cell injury in HGECs *in vitro*, whereas 1-MH protected HGECs from injury caused by high glucose.

The coincidence of retinal and nephropathy pathology in diabetic patients is recognized, and the definition of “renal-retinal syndrome” has been proposed in the literature (52). In diabetes, endothelial dysfunction occurs in both the retina and kidney, indicating that there might exist potential factors inducing endothelial cell damage in the circulation. Exosomes carrying a variety of proteins and RNAs are vesicles actively secreted by cells (14–16). Exosomes play a role in cell communication, cell migration, tumor cell development, and angiogenesis (14–16). Exosomes abundant in serum have multiple activities, such as remodeling the ECM and releasing the contents into recipient cells to deliver signals and molecules to target cells and organs (16). Differentially expressed proteins and metabolites in HGECs incubated with SExos from DR and DN patients were discovered in this study, demonstrating that SExos can mediate changes in the expression of proteins and related metabolites in endothelial cells, affecting the function of the microvascular endothelial barrier in diabetes.

Under physiological conditions, microvascular endothelial cells mainly exert anticoagulant activity (53, 54). When microvascular endothelial cells are damaged, they lose their coagulation activity and exert procoagulant activity (53–55). Fibrinogen is a critical coagulation factor that plays a role in diabetic vascular disease, and fibrinogen synthesis is increased in T2D (56, 57). Furthermore, hyperfibrinogenemia is a marker for inflammatory alterations that result in endothelial dysfunction (56, 58, 59). According to this research, despite correcting for a range of factors in type 2 diabetes mellitus (T2DM), serum fibrinogen and DR show an independent connection, suggesting that they may play a role in the development of diabetic microvascular issues (60). There was a significant connection between retinopathy and serum fibrinogen level in DM participants after adjusting for all traditional indicators of atherosclerosis except serum creatinine (61). When serum creatinine was added to the other markers, the association was attenuated (61). In T2D individuals with higher urine albumin excretion, fibrinogen is upregulated and albuminuria is linked to increased fibrinogen and albumin production (62, 63). Plasma fibrinogen is required for the regulation of the inflammatory response, and high levels of plasma fibrinogen are linked to increased inflammation (64, 65). Plasma fibrinogen is a strong predictor of DN in T2D patients, according to earlier research. Fibrinogen is a protein that is released in the acute phase of infection or systemic inflammation (66–68). Furthermore, it is widely known that total fibrinogen levels rise during inflammation (68). Many studies have found that patients with diabetic microvascular problems had high plasma fibrinogen levels and that plasma fibrinogen levels are associated with the severity of DR (60). In this study, the proteomic profiles and PRM results showed that FIBA was significantly upregulated in HGECs cultured with DR+DN patients’ SExos. WB results confirmed that FIBA was significantly increased in HGECs incubated in high glucose, consistent with the trend in previous studies.

Metabolite 1-MH is a biological derivative of the dipeptide anserine, classified as a methylamino acid, which is abundantly presented in muscles (69). 1-MH was reported to be associated with the increased rate of chronic kidney disease (CKD) (70). In a previous study, 1-MH was measured to be decreased in the plasma of DM (71). In our study, the content of 1-MH in HGECs treated with SExos from DR+DN patients was significantly reduced. The latest study indicated that the visceral adipose tissues of metabolic syndrome presented a decreased 1-MH compared with non-obese individuals (72). The level of urinary 1-MH of patients with arterial hypertension is recognized to decrease (73). High 1-MH concentration in the urine is independently associated with the lower risk of graft failure in kidney transplant recipient, (KTR) (74). In our study, 1-MH could reverse the HGEC injury induced by high glucose, demonstrating the protective effect of 1-MH on endothelial cells under a high glucose environment.

The correlation network of differentially expressed proteins and differential metabolites indicated that SODM, OPLA, and ITB3 presented positive correlations with 1-MH, while FIBA presented negative correlations with 1-MH. However, the pathway between 1-MH related to FIBA that affects the diabetic microvascular endothelial barrier still needs further verification, which we will conduct in the follow-up experiments. In our joint bioinformatics analysis results, the MSEA pathway enrichment result showed that the cysteine and methionine metabolism might play an essential role in the pathogenic process of diabetic endothelial dysfunction. It has been reported that, compared to the human donors from the age-matched non-diabetic people, the retina from DR patients presented up to 3-fold higher homocysteine levels, and the key enzymes that are important for cysteine metabolism show 40%–60% lower levels in the retinal microvasculature from DR donors (75). By constricting vessels, modifying blood coagulant characteristics, causing oxidative harm to the vascular endothelium, and damaging the arterial walls, abnormal cysteine metabolism can induce cardiovascular disorders (76). Homocysteine levels in diabetes are thought to be a biomarker for microvascular problems such as diabetic neuropathy, retinopathy, and nephropathy (76). Elevated oxidative stress and reactive oxygen species (ROS), which produce glomerular endothelial dysfunction, result in a change in GFR, which causes renal dysfunction as seen by increased homocysteine and cysteine, according to studies (77, 78). Moreover, previous studies reported the importance of the KEGG pathway taste transduction in the pathogenic process, which addresses our attention. Hispanic individuals with lower carotid plaque have different allele frequencies for single-nucleotide polymorphism (SNPs) within the taste receptor genes compared to those of Hispanic individuals with a more significant plaque burden (79). The sweet taste receptors were reported to be involved in the activation of ROS-NLR family pyrin domain containing 3 (NLRP3) inflammasome signaling in the pathogenesis of DN, suggesting that the taste transduction pathway might be the potential pathogenic pathway in DN (80). Pulmonary microvascular barrier dysfunction could be alleviated by activating the sweet taste receptor (81).

However, some limitations must be considered when interpreting the outcomes of this study. The first is the metabolomics and proteomics analyses’ sample size constraint. The second constraint is the link between 1-MH and FIBA, which necessitates the use of additional metabolomics methods to quantify and verify small-molecule metabolites. In the future, we will further explore the roles of FIBA and 1-MH in the microvascular endothelial barrier of diabetes by using transfection technology, high glucose-incubated endothelial cell model and streptozotocin (STZ)-induced rat model. To investigate the damage effect and mechanism of SExos from DR+DN patients on endothelial cells, *in vitro* studies, metabolomics, proteomics, and bioinformatics were combined. From the results, it can be confirmed that patients’ SExos might cause endothelial dysfunction mainly by upregulating FIBA and downregulating 1-MH; FIBA correlating to 1-MH might be through the pathway of reducing the excessive cysteine and methionine metabolism. Taste transduction could possibly play a role.

Overall, our findings suggest that FIBA overexpression and 1-MH loss may be linked to the pathogenicity of diabetic endothelial dysfunction in DR/DN, implying that a cohort study is needed to further investigate the role of FIBA and 1-MH in the development of DN and DR, as well as the related pathways between the two proteins.

## Data Availability Statement

The original contributions presented in the study are publicly available. These data can be found here: ProteomeXchange, PXD030660.

## Ethics Statement

The studies involving human participants were reviewed and approved by the Clinical Trial Management System of the First Affiliated Hospital of Zhengzhou University. The patients/participants provided their written informed consent to participate in this study. Written informed consent was obtained from the individual(s) for the publication of any potentially identifiable images or data included in this article.

## Author Contributions

JY implemented the study, collected the data, analyzed the data, and wrote the article. JY, DL and ZL participated in the design and interpretation of the studies, analysis of the data, and review of the article. All authors contributed to the article and approved the submitted version.

## Funding

This work was supported by the National Natural Science Foundation of China (General Program–81970633), Natural Science Foundation of Henan Province (202300410363), National Natural Science Foundation of China (81800648), and Major Science and Technology Special Project of Henan Province (201300310600).

## Conflict of Interest

The authors declare that the research was conducted in the absence of any commercial or financial relationships that could be construed as a potential conflict of interest.

## Publisher’s Note

All claims expressed in this article are solely those of the authors and do not necessarily represent those of their affiliated organizations, or those of the publisher, the editors and the reviewers. Any product that may be evaluated in this article, or claim that may be made by its manufacturer, is not guaranteed or endorsed by the publisher.
